# From Policy to Politics: Is there a Nordic Model for the regulation of alcohol and gambling?

**DOI:** 10.1177/14550725251350445

**Published:** 2025-07-01

**Authors:** Virve Marionneau, Mikaela Lindeman, Jenny Cisneros Örnberg, Thomas Karlsson

**Affiliations:** The Centre for Research on Addiction, Control and Governance, 3835 University of Helsinki, Helsinki, Finland; Promotional and Preventive work unit, Finnish Institute for Health and Welfare, Helsinki, Finland; Department of Public Health Sciences, Centre for Social Research on Alcohol and Drugs, Stockholm University, Stockholm, Sweden; Promotional and Preventive work unit, Finnish Institute for Health and Welfare, Helsinki, Finland

**Keywords:** alcohol, best buy, gambling, Nordic comparison, Nordic model, policy, policy outcomes

## Abstract

**Aim:** Control policies in the fields of alcohol and gambling show similarities across Nordic countries. The so-called Nordic model has consisted of strong state intervention and, in many cases, monopolistic provision. However, internal and external pressures pose challenges to the model. This article provides a comparative review of current policies on alcohol and gambling in the Nordic region. **Methods:** We analyse framings, implementation and outcomes of current alcohol and gambling policies in Denmark, Finland, Iceland, Norway and Sweden. We produce a review of legal documents and grey literature. We also analyse on-going and recent policy changes (2021–2024). Our focus is on policies related to World Health Organisation's evidence-based “best buys”: physical availability, taxation and marketing. While these best buys are designed for alcohol, we also apply these to gambling. **Results:** Control policies in the region take place on a spectrum from more restrictive to more market-oriented. Overall, alcohol policy is more restrictive than gambling policy. In comparison with gambling policy, alcohol policy was characterised by a stricter public health framing and consistent implementation based on the best buys. In terms of policy outcomes, more stringent alcohol regulations translated, to some extent, to lower consumption and prevalence, but not systematically. Recent policy developments suggest that alcohol policy may be heading towards increased de-regulation, whereas gambling policies are becoming stricter. **Conclusions:** The Nordic model continues to be visible particularly in alcohol control. However, the model is challenged by politics. To persist, the Nordic model needs political will and increased cross-country and cross-sectoral collaboration.

## Introduction

The Nordic countries (Denmark, Finland, Iceland, Norway and Sweden) have many commonalities in terms of history, values and societal frameworks. Nordic countries also share many features when it comes to control policies. The so-called “Nordic model” or “Nordic way” has been described as a set of common traits in terms of policy objectives and policy designs within the region (Andersen et al., 2016). The Nordic model is defined by policies that align with principles of the welfare state. In economic policy, the Nordic model involves combining state control with free-market capitalism (Andersen et al., 2007). Policy approaches to the sale of addictive commodities have also been characterised by state control. In most Nordic countries, regulation and control of alcohol and gambling, have traditionally focused on active state intervention in the market and related restrictions on private profit-seeking. The idea of the Nordic model has been used to understand Nordic alcohol policy, in particular (Room & Tigerstedt, 2007). The term has also been used to analyse gambling policies and cultures in the Nordic region (Matilainen, 2016; Thompson, 2025).

In terms of concrete policy tools, the Nordic model of regulating alcohol and gambling has involved stringent restrictions on physical availability and marketing, as well as high price levels via heavy taxation. Restrictions on physical availability have been embodied by state monopolies in most of the Nordic countries. Historically, and still today, monopolies have been used as a vehicle for the double purposes of public health as well as state revenue generation (Room & Cisneros Örnberg, 2019). Monopolies in the alcohol and gambling markets have been justified in terms of the harms and costs to individuals and societies (Babor et al., 2023; Sulkunen et al., 2021). In the Nordics, policies have therefore aimed at protecting public health with effective population-level regulation (Room & Cisneros Örnberg, 2019; Room et al., 2006). In addition to their public health mission, monopolistic operators have been charged with public finance objectives to produce revenue for states (Room & Cisneros Örnberg, 2019; Nikkinen & Marionneau, 2021).

The tradition of strong state control is still visible today in how alcohol and gambling are regulated in the Nordic region. All Nordic countries, apart from Denmark, continue to restrict the sale of alcohol within monopolistic structures (Jónsson & Kristjánsson, 2013; Tigerstedt, 2001; Karlsson et al., 2020; Karlsson, 2014). Even the self-governing Faroe Islands operate their own alcohol monopoly, founded in 1992 (Larsen & Hansen, 2023). Until the 2010s, the regulation and provision of gambling has also been organised via monopolistic structures in most Nordic countries. However, regulatory changes have since been implemented in Denmark, Sweden and, currently, in Finland (Nikkinen & Marionneau, 2021; Spångberg & Svensson, 2022; Cisneros Örnberg & Tammi, 2011; Cisneros Örnberg & Hettne, 2018; Järvinen-Tassopoulos et al., 2024).

In recent years, the Nordic model has been challenged by both internal and external pressures. Globalisation of markets and the free circulation of goods and services within the European Union (EU) and the European Free Trade Association (EFTA) areas have challenged the possibilities of the monopoly model to restrict consumption (Marionneau & Hellman, 2020; Örnberg & Ólafsdóttir, 2008). At the same time, monopolies are challenged by strong commercial interests in the provision of these products (Marionneau & Hellman, 2020). In the gambling field, monopolies have faced competition from so-called offshore providers in online environments. Imports and online trade are also challenging the premise of alcohol monopolies (Trolldal, 2023; Finnish Institute for Health and Welfare, 2024a). Further competition emanates from within-borders in the form of farm sales or expanded alcohol availability in retail networks (Lindeman, 2024; Dahlström, 2024).

The evolving landscape of alcohol and gambling regulation raises the question whether a ‘Nordic model’ still exists in the field. The aim of this study is to provide a comparative review of current policies and policy debates on alcohol and gambling across the Nordic region. We analyse how current policies are formulated, implemented, how they have developed in relation to consumption and harm indicators, and how policies are changing. Our findings shed light on the regulatory systems and practices governing alcohol and gambling in Nordic countries and on the future prospects of the Nordic model.

## Methods

### Theoretical premise

We produced a review of current gambling and alcohol policies in the Nordics by systematically scoping for relevant documents. The aim was to analyse objectives, implementation and outcomes of Nordic policies on alcohol and gambling to understand current similarities and differences in policy within the region. We also analysed on-going political debates to change policy to gain understanding of policy directions. In line with the scoping review methodology applied to policy studies, we therefore focused on mapping rather than assessing policies (Peters et al., 2021).

Our analysis is informed by theoretical understandings of cyclical policy processes. Policy cycles are a heuristic tool used in public policy literature to understand the different phases of policy processes. Policy cycles consist of distinct policy phases, including agenda-setting (framing), formulation, adoption, implementation and evaluation (Jann & Wegrich, 2017; Lasswell, 1956). Understanding policy processes as cycles emphasises the importance of feedback loops between phases and the perpetuation of the process (Jann & Wegrich, 2017). A focus on the wider policy life cycle provides a more comprehensive view of policy directions. Because policy processes are cyclical, they are also in a constant process of renewal (Lasswell, 1956). Most gambling policy research focuses on individual policy phases (cf. Aimo et al., 2023). Conversely, Nordic alcohol research has traditionally focused on longer-term trends and processes (Tigerstedt, 2001; Ugland, 2002; Cisneros Örnberg, 2009; Karlsson, 2014).

In the current analysis, we focus on three crucial stages of the policy process: agenda setting (how policies are framed and what their stated aims are), implementation (what kind of policy tools are available to reach these aims) and evaluation (potential outcome indicators). In addition, we analyse on-going policy changes.

### Data and search strategy

As a result of our focus on three distinct policy phases (framing, implementation and outcomes), as well as policy changes, our data retrieval strategy consisted of several steps. First, agenda setting and implementation were analysed based on current legal provisions. We conducted searches for the main gambling or alcohol legislation from national legal databases (Denmark: Retsinformation; Iceland: Government website; Finland: Finlex; Norway: Lovdata; Sweden: Riksdagen). The searches were conducted during August to September 2024 and reflect the situation at that time. This dataset included up-to-date legislation on alcohol and gambling in each Nordic country. This dataset allowed us to identify legal framings and implementation of policies.

Second, evaluation (potential outcome indicators of policies) was analysed using key consumption indicators related to total consumption and harm. We retrieved these from national public health and gambling authorities or statistical repositories. We included the latest data available. In addition, we contacted national agencies and departments, as well as academics, familiar with gambling or alcohol policy to gain further information. All data in other currencies than Euro were converted into Euro using the conversion rate of 2 December 2024. For per capita figures, we divided available total sales figures by total population over 18 years old.

Third, we scoped additional sources to identify any on-going alcohol or gambling policy changes and policy debates that are not yet visible in up-to-date legislation. For alcohol policy, we scanned relevant information and news on two specialised websites: Nordic alcohol (www.nordicalcohol.org) and PopNAD (www.popnad.com). Nordic alcohol is the website of the Nordic alcohol and drug policy network (Nordan) that regularly posts information and news on these topics based on debate in large Nordic newspapers and newsletters. PopNAD is a Nordic popular scientific website that publishes articles on the alcohol, narcotic, doping, tobacco and gambling fields. We conducted a comprehensive search of the Nordic Alcohol and Drug Policy Network (NordAN) website using the keyword “alcohol policy”. The search yielded 218 entries from January 2021 to November 2024. From this dataset, we selected entries specifically pertaining to the Nordic countries. Additionally, we reviewed all 48 articles published under the “alcohol” category on the PopNAD platform to ensure comprehensive coverage and to avoid any omissions in our analysis.

To analyse gambling policy changes, we charted news on policy developments in the region using the database of Vixio Gambling Compliance (2021). Vixio is a gambling intelligence service providing news on regulatory and market developments, under license. To capture all on-going and recent policy debates in the field, we searched for any news on policy changes in the Nordic countries between January 2021 and September 2024 using the regulatory updates section of the database. We first retrieved all regulatory updates on the Nordic countries (*N* = 349). However, most of these concerned fines issued to operators for various breaches, regulatory improvements to Anti-Money-Laundering policy, or policy changes in Finland focusing on improving control of the national monopoly (prior to 2023) or shifting the regulatory model to a licensing system (late 2023 and 2024). We only included regulatory changes that focused on availability, taxes, visibility or other gambling-specific consumer protection measures (*N* = 26). Due to lack of reporting on Iceland in the Vixio database, we also conducted web scraping to find other sources on Icelandic gambling. This resulted in the inclusion of one additional source, as reported in the results.

### Analysis

Legal framings were retrieved from legislative framing sections of national alcohol and gambling laws. These legislative intents are usually presented in the first paragraphs of legal documents. Legislative framings are an indication of the overall principles governing policy and the main aims of its implementation. For the analysis, we classified these framings thematically based on recurring categories and themes.

Policy implementation-related information was retrieved using the World Health Organisation's (WHO) “best buys” approach. The WHO best buys relate to successful regulation of alcohol policy and include three key regulatory tools: restricting physical availability, high taxes (restricting affordability) and restricting marketing (WHO, 2011; Babor et al., 2023). While all of these policies can serve multiple functions, they have concrete public health implications. To enable comparison, we also used the same best buy indicators for gambling policy, although these are less established within that field. In addition to best buys, we included information on the regulating authorities because these were expected to influence regulatory prioritisation.

There were some limitations in using the same analytical framework to compare alcohol and gambling. Gambling is increasingly a digital-first commodity (Wardle et al., 2024), whereas alcohol is a tangible substance. For this reason, we did not include information on opening hours in our analysis despite their importance in alcohol regulation. Conversely, we have also not included policy initiatives to block and control offshore markets as these are highly gambling-specific.

Policy outcomes were analysed using key regulatory and consumption indicators. We constructed a framework to analyse these indicators inductively, based on an initial reading of our data and the possibilities of comparison. The outcome indicators focused on total consumption-related indicators, including per capita consumption, financial surplus to state, and population prevalence of use and problematic use.

Finally, we compared ongoing and recent policy changes in the gambling and alcohol sectors using the same best buy categories as in our analysis of implementation. The choice to focus only on these categories was our overarching aim at mapping public health-related policies rather than the full policy development field (including also other regulatory priorities such as crime prevention, anti-money laundering, or data protection issues, see Wardle et al., 2024). Because the best buys are not established in the gambling sector, we also included a category for “other consumer protection measures”. This allowed us to capture potential additional developments in gambling regulation.

## Results

### Legal framings

Framings set the aims of policies. A summary of the legislative framings given in alcohol and gambling policies across the Nordic countries is provided in Table 1. Results show divergence across framings for alcohol and gambling: In each Nordic country, alcohol laws aim at reducing population-level harm and damage by limiting total consumption. For gambling, legislative framings are more varied. Population-level harm prevention was also one of the legislative aims for gambling control in all Nordic countries. However, the vocabulary differed from alcohol legislation. In alcohol laws across all countries, harm prevention included limiting total consumption. For gambling, harm prevention did not include references on limiting total consumption, but rather on limiting the negative consequences of consumption.

**Table 1.** Legislative framings of alcohol and gambling policies in Nordic countries.

	Limiting harm and/or consumption	Security, legality and crime prevention	Financial reasons
**Denmark**			
Alcohol	No unified act, no over-arching goal		
Gambling	Keep consumption at moderate level; Protect youth and vulnerable populations	Fair, responsible, and transparent provision; Prevent crime	
**Finland**			
Alcohol	Minimising harm and consumption		
Gambling	Prevent and reduce economic, social, and health harms	Legal protection; Prevent crime	
**Iceland**			
Alcohol	Minimising harm and consumption; improve public health and social responsibility; Protect youth		
Gambling	Prevent harmful effects on the public	Maintain public order	Raise funds for the public good
**Norway**			
Alcohol	Minimising harm and consumption		
Gambling	Prevent gambling problems and other negative consequences	Responsible and safe provision	Direct profits to non-profit purposes
**Sweden**			
Alcohol	Minimising harm and consumption		
Gambling	Limiting negative consequences, high consumer protection (duty of care)	High security, Prevent crime	Public benefit purposes

Sources: Denmark: Spilleloven (2020); Finland: Alkoholilaki (2017), Arpajaislaki (2001); Iceland: Áfengislög (2011), Lög um happdrætti (2005); Norway: Alkoholloven (1989); Pengespilloven (2022); Sweden: Alkohollag (2010), Spellag (2018).

Another key difference in legislative framings for alcohol and gambling related to the number of aims. In all Nordic countries, except for Denmark, harm prevention and limiting total consumption was the sole aim of alcohol legislation. While harm prevention was also included in each gambling law, this framing was presented alongside a range of other aims such as crime prevention, security or revenue generation. For example, in Iceland, the gambling law included an aim to raise funds for public good, including “social, cultural, sports, or charitable causes, such as international humanitarian work” (Law on Lotteries, No. 38/2005, 2–3 gr.).

We also found differences between countries, particularly with regard to gambling legislation. The breadth and scope of public health reasoning varied across countries. In Finland, Iceland and Norway, the legislative aim was to *prevent* harm from gambling, while, in Sweden and Denmark, the aim was only to limit or reduce harm. Similarly, countries differed in how many legislative aims were included. Sweden, Norway and Iceland included aims across all three categories (harm, safety and financial), whereas financial reasons were not part of the legislative aims in Finland or Denmark.

### Policy implementation

Table 2 presents the main responsible authorities in charge of implementing alcohol and gambling policy. The results show important divergence in terms of regulatory responsibility for alcohol and gambling. All Nordic countries entrust the regulation of alcohol-related matters to ministries responsible for health or social affairs. Iceland has split the responsibility between the Ministry of Health, which is in charge of public health policies, and the Ministry of Finance, which is responsible for setting and collecting taxes on alcohol products. In contrast, health ministries are not in charge of the regulation of gambling in any Nordic country. Instead, regulation of gambling is undertaken by ministries in charge of taxation and finance (Denmark and Sweden), interior (Finland), justice (Iceland) or culture (Norway). This result reflects the overall aims of the legal frameworks.

**Table 2.** Responsible ministries and authorities for alcohol and gambling control in the Nordics.

	Responsible Ministry	Responsible Authority
Denmark		
Alcohol	Ministry of the Interior and Health	Danish Health Authority
Gambling	Ministry of Taxation	The Danish Gambling Authority
Finland		
Alcohol	Ministry of Social Affairs and Health	Regional State Administrative Agency
Gambling	Ministry of the Interior	Administration at the National Police Board
Iceland		
Alcohol	Ministry of Health; Ministry of Finance and Economic Affairs	Icelandic Alcohol and Tobacco Commission
Gambling	Ministry of Justice	n/a
Norway		
Alcohol	Ministry of Health and Care Services	The Norwegian Institute of Public Health
Gambling	Ministry of Culture and Equality	Norwegian Gambling and Foundation Authority
Sweden		
Alcohol	Ministry of Health and Social Affairs	Public Health Agency
Gambling	Ministry of Finance	Gambling Authority; Public Health Agency

Sources: Denmark: Spilleloven (2020); Finland: Alkoholilaki (2017), Arpajaislaki (2001); Iceland: Áfengislög (2011), Lög um happdrætti (2005); Norway: Alkoholloven (1989), Pengespilloven (2022); Sweden: Alkohollag (2010), Spellag (2018).

n/a = not available.

The responsible authorities for the implementation of policy and regulation also varied across countries. Overall, duties related to gambling regulation were given to specialised regulatory bodies, whereas alcohol regulation was undertaken by public health administration. For example, in Denmark and Sweden, national public health agencies are also tasked with developing guidelines and running public health campaigns to reduce alcohol harm.

Table 3 presents the results of policy implementation based on WHO best buys. We noted the regulatory model and number of licenses (availability indicators), marketing restrictions and tax levels (pricing indicator). Similarly to policy framings, alcohol policy implementation was stricter than the implementation of gambling policy. This was visible particularly as somewhat higher age limits for some alcohol products (with the exception of Denmark), higher prevalence of monopolistic structures for alcohol (again, with the exception of Denmark) and stricter rules for alcohol marketing in some countries. Tax levels could not be compared between alcohol and gambling products due to highly differing tax mechanisms and differences in reporting tax.

**Table 3.** Regulatory indicators for alcohol and gambling control in the Nordics.

	Regulatory model	Number of licenses	Age limits	Marketing restrictions	Tax levels*
Denmark					
Alcohol	Licensing	Private resale network	18 years (except 16 years for alcohol under 6% off-premises)	Allowed, but no marketing to youth.	Beer: 655 €/HLPA; Wine: 1,375 €/HLPA; Spirits: 2,016 €/HLPA
Gambling	Licensing, except for state monopoly on lotteries and some forms of betting	25 online betting, 39 online casino (2022), land-based private resale network	18 years	Allowed, but no marketing to youth, no misleading content.	28%/GGR
Finland					
Alcohol	State monopoly for fermented beverages over 8%	372 Alko shops (2023), private resale network	18 years max 22%; 20 years over 22%; 18 years for all alcohol on-premises	Allowed only for beverages under 22%. Restrictions on content and placement, no targeting youth.	Beer: 3,850 €/HLPA; Wine: 3,827 €/HLPA; Spirits: 5,035 €/HLPA
Gambling	State monopoly	Online monopoly, land-based private resale network	18 years	Allowed only for less harmful products. Restrictions on content, no targeting youth.	Monoply profits to state and gambling tax 5%/GGR
Iceland					
Alcohol	State monopoly	50 ÁTVR shops (2023)	20 years	Prohibited	Beer: 4,367 €/HLPA; Wine: 5,753 €/HLPA; Spirits: 9,787 €/HLPA
Gambling	Monopolies by product range	Monopoly	18 years	Allowed for monopolies.	Monopoly profits to beneficiaries
Norway					
Alcohol	State monopoly for beverages over 4.7%	350 Vinmonopolet shops (2023); private resale network	18 years max 22%; 20 years over 22%; 18 years for all alcohol on-premises	Prohibited	Beer: 4,364 €/HLPA; Wine: 4,364 €/HLPA; Spirits: 7,435 €/HLPA
Gambling	State monopoly	Online monopoly, land-based private resale network	18 years	Allowed only for less harmful products. Restrictions on content, no targeting youth.	Monopoly profits to beneficiaries
Sweden					
Alcohol	State monopoly for beverages above 3.5%	452 Systembolaget shops (2023); private resale network	18 for resale network and on-premises, 20 years for monopoly	Allowed only for beverages below 15%, restrictions on content, no targeting youth	Beer: 1,927 €/HLPA; Wine: 2,270 €/HLPA; Spirits: 4,927 €/HLPA
Gambling	Licensing, some products under state monopoly	64 commercial online betting and gambling licenses (2024)*, private resale network	18 years; 20 years for land-based casino	Allowed but should be ‘moderate’ and not aimed at youth.	22%/GGR, gambling for public benefit purposes exempted (Lag (2024:276)

Sources: Denmark: Spilleloven (2020), Spillemyndigheden (2022); Finland: Alkoholilaki (2017), Arpajaislaki (2001, updated); Iceland: Áfengislög (1998), Lög um happdrætti (2005), Áfengislög 75/1998; Norway: Alkoholloven (1989), Pengespilloven (2022); Sweden: Spellag (2018), Alkohollag (2010); General: Vixio Gambling Compliance (2021) country profiles, Spirits Europe key data.

GGR = gross gambling revenue; HLPA = hectolitre of pure alcohol.

*All figures for alcohol taxation are from 2021.

**Figures do not include charity licenses.

We also found differences between the Nordic countries. Denmark stands out as a more “liberal” country with regard to both alcohol and gambling policy. There is no state monopoly on alcohol, and the gambling monopoly only covers a minority of products (lotteries and some land-based sports betting). Other Nordic countries operated resale monopolies for the sales of alcoholic beverages, although to differing thresholds. In Iceland, the state monopoly covers all alcoholic drinks. In other monopoly countries, Finland had the highest threshold for alcoholic beverages that could be sold outside of the monopoly structure (8% compared to 4.7% in Norway and 3.5% in Sweden).

For gambling, state monopolies were operated for some products in all countries. In Norway, Finland and Iceland, all regulated gambling offers were provided under monopolistic umbrellas. However, the resale of many gambling products is also allowed for private retailers, such as supermarkets or restaurants. This was the case for products such as scratch cards, lotteries and, to some extent, electronic gambling machines (EGMs).

Marketing regulations varied to some extent across countries and products. Norway and Iceland prohibit marketing of alcohol. In Finland and Sweden, marketing is only permitted for products under 22% or 15%, respectively. All countries also prohibit targeting advertising at young audiences. Content is regulated to not encourage an overly positive image of alcohol use. For example, in Finland, the Alcohol Act included several rules on alcohol marketing, such as controls on content, media, public visibility, sponsorship and digital marketing in social media. In Sweden, all alcohol advertisements must also include a health warning.

Gambling advertising is permitted in all countries, except Iceland. All countries have limitations on the content and target audiences of gambling advertising (youth and vulnerable populations). There are also some restrictions on which products can be advertised in Finland and Norway. Both Finland and Norway also state that marketing of gambling should aim at “channelling” consumption from offshore markets to the monopoly.

In terms of taxation, Iceland and Norway had the highest levels of overall alcohol tax, whereas tax levels in Denmark were comparatively lower than in other Nordic countries. In all countries, tax levels for alcohol beverages varied depending on the type of alcohol. Beer or wine are generally taxed at the lowest levels, while the tax rates for spirits are higher. Alcohol taxation is typically counted as euros per hectolitre (100 litres) of pure alcohol (HLPA). For gambling, taxation of licensed operators is based on so-called gross gambling revenue (GGR) (i.e., company revenue after winnings have been paid out). Tax levels in Denmark are higher than in Sweden (28% compared to 22%). In monopolistic countries, gambling profits are transferred to state coffers (Finland) or to earmarked beneficiaries (Norway and Iceland).

### Policy outcomes

We also analysed potential outcomes of policy choices. The focus of this part of the analysis was on consumption indicators, including sales, per capita consumption and population prevalence. The results are described in Table 4. The results show some divergence across countries and products. Many indicators are difficult to compare between gambling and alcohol as a result of differing levels of monitoring between commodities and across countries. Overall, we found that the population-level prevalence of alcohol consumption tends to be higher than gambling consumption. We also found that, in all countries, gambling and alcohol are significant to national economies in terms of surplus transfers to states. In most contexts, surplus to state from alcohol is higher than from gambling, reflecting higher total consumption.

**Table 4.** Consumption indicators of alcohol and gambling in the Nordics (2022/2023).

	Per capita consumption/GGR (euro)	Surplus to state	Population prevalence (12 months, adults)	Population prevalence at risk use (12 months)
Denmark				
Alcohol	9.3 l/18+	0.523 bn €	92–94%	20% (problematic use)
Gambling	288 €/18+	0.332 bn €	63%	4.3% (2021 PGSI 3+)
Finland				
Alcohol	8,7 l/15+	1.463 bn €	88%	28% (AUDIT any score)
Gambling	225 €/18+	0.63 bn €	70%	4.2% (2023; PGSI 3+)
Iceland				
Alcohol	7.7 l/15+	n/a	80%	25% (estimate)
Gambling	n/a	n/a	n/a	0.7% (PGSI 8+)
Norway				
Alcohol	6,83 l/15+	0.545 bn €	90%	5–8% (problematic use)
Gambling	200 €/18+	0.652 bn €	62%	2.3% (PGSI 3+)
Sweden				
Alcohol	8,6 l/15+	1.527 bn €	82%/16–84 year	16% (hazardous use)
Gambling	280 €/18+	0.234 bn €	56%/16–84 year	1.3% (PGSI 3+, 2021)

Denmark: Spillemyndigheden (2023); Danmarks statistik (2023); Bloomfield et al. (2022); Statens Institut for Folkesundhed (2024); Skatteministeriet (2024).

Finland: Veikkaus (2024); Finnish Institute for Health and Welfare (2024b, 2024c); Mäkelä and Warpenius (2024).

Iceland: Statistics Iceland (2023); Kristjánsson et al. (2023); Dómsmálaráðuneytið (2024); Viðskiptaráð (2024).

Norway: Folkehelseinstituttet (2022, 2023); Pallesen et al. (2023); Norsk Tipping (2024); Norsk Rikstoto (2024); Ministry of Finance (e-mail, 2024).

Sweden: Spelinspektionen (2024); Nationella folkhälsoenkäten (2024); CAN (2023); Folkhalsomyndigheten (2023).

General/Multiple Countries: Nordic Welfare Center (2024); World Health Organization (n.d.); Tran et al. (2024).

GGR = gross gambling revenue; n/a = not available; PGSI = Problem Gambling Severity Index.

Where differences between countries could be compared, we found that the more market-oriented policy implementation in Denmark, and to a lesser degree in Finland, is reflected as higher population prevalence of alcohol use and somewhat higher per capita consumption of gambling in monetary terms. Norway has the overall lowest levels of per capita consumption on alcohol and gambling. However, the population prevalence of alcohol use is high. Levels of problematic alcohol use are difficult to compare because of the widely varying measures used in reporting in the region. For gambling, all countries use the Problem Gambling Severity Index (PGSI) in population studies. These show that Finland and Denmark have the highest prevalence rates of potential gambling problems (PGSI 3+). Finland also has the highest population prevalence of past year gambling participation.

### Current policy changes and debates

The final part of our analysis focused on recent and on-going policy changes in the alcohol and gambling fields of the Nordic countries (2021–2024). The analysis is based on best buy indicators (availability, taxation and marketing). Furthermore, we included other possible consumer protection measures that had been taken. The results for the policy change analysis in the alcohol field are presented in Table 5.

**Table 5.** Alcohol policy changes in the Nordics (2021–2024).

	Availability	Taxation and pricing	Marketing	Other consumer protection
Denmark	Harm prevention plan with raise in age limits from 16 to 18 years for some mild beverages (2024)			
Finland	Alcoholic beverages up to 8% permitted in resale network (2024); Inquiry into permitting wine up to 15% in resale network (2024); Plan to permit home deliveries and online sales (2024). The Government will allow all Finnish microbreweries, craft breweries, micro distilleries and vineyards to sell their products directly to consumers from their production sites under a retail trade licence	Tax hikes for spirits and wine, tax decrease for beer (2024), Further plans to increase tax on spirits (2025)		Discussion on moving responsible ministry from Ministry of Social Affairs and Health to Ministry of Economic Affairs and Employment
Iceland	Farm sales, direct sales to consumers from (2022)	Tax increases over the years		
Norway		Moderate tax increases (inflation checks)		Regulator given powers to fine for misconduct (2024)
Sweden	Suggestion to permit farm sales of alcohol (2025), Evaluation of Alcohol Policy was launched in 2024, specifically examining the effects of different alcohol policy instruments to ensure they fulfill their public health objectives	Moderate tax increases (inflation checks), tax reduction for microbreweries and small breweries (2025)	Suggested prohibition of promoting retail sales and private imports (2024)	

Sources: Nordic alcohol database; PopNAD database.

Most alcohol policy changes have related to availability and taxation. Most tax-related policy changes were tax increases. However, in Finland and Sweden, tax levels for beer have also been, or are planned to be reduced.

Availability policies vary. In Finland, Iceland and Sweden, some availability restrictions have been lifted or are projected to be lifted to varying degrees. Availability-related changes have been the most significant in Finland, with the permission for private retailers to sell fermented alcoholic beverages of up to 8% alcohol by volume (ABV) as of 2024. An on-going inquiry is also looking into permitting private retailers to sell alcoholic beverages of up to 15% ABV. In Denmark, availability was restricted for youth, with age limits raised for off-premises purchases for milder beverages. In practice, this means prohibiting sale of alcohol products with >6% ABV to adolescents aged 16–17 years, instead of the previous 16.5% ABV limit. However, the new age limit still allows 16–17-year-olds to buy milder alcohol products, such as beer and cider.

The results for the policy change analysis in the gambling field are available in Table 6. Unlike for alcohol, we identified policy changes across all best buys for gambling. Also unlike for alcohol, most changes relating to availability are aimed at restricting rather than increasing availability. The contrast is starkest in Finland. Although alcohol availability has been increased in Finland, the availability of gambling has been restricted during the period via the introduction of mandatory identification, as well as restrictions to opening hours of land-based EGMs. However, this trend may change with the advent of the licensing system, set to become operational in 2027.

**Table 6.** Gambling policy changes in the Nordics (2021–2024).

	Availability	Taxation and pricing	Marketing	Other consumer protection
Denmark	Mandatory identification for retail betting (2023); Legalisation of new online products (bingo, animal race betting) (2021)	Annual fee for EGMs increased (2023, 2022); Increase in gambling taxes 20%→28% (2021)	Proposed restrictions on advertising including restrictions on time slots, use of celebrities, marketing of bonuses (2022)	Deposit limits (2023)
Finland	Announced reform of online gambling to licensing in 2026/2027; Payment service provider blocking (2022); Restricted EGM operating hours 9 am to 9 pm (2022); Mandatory identification for land-based gambling (2022); Closure of one land-based casino (2024)	Lottery tax reduced (5.5%→3.4% in 2022 and 5.0% in 2023)		Lower loss limits for identified gambling (2021); Compulsory loss limits for EGMs (2021); Duty of care for Veikkaus (2022)
Iceland	Discussion on licensing system (2024)			
Norway	DNS blocking of offshore websites (2025); proposal to abolish monopoly rejected (2021)		Television advertising prohibited (2022)	Stricter loss limits on under 20-year-olds (2023)
Sweden	“Safer gambling proposal” with provisions for b2b licensing to prevent unlicensed gambling (2023); Closing of land-based casinos 2024)	Ban on gambling on credit (2024); Increase in gambling tax rate 18%→22% (2024)	“Safer gambling proposal” with provisions for extended marketing restrictions for unlicensed gambling (2023)	Strengthening duty of care via increased access to consumer data for companies (2023)

Source: Vixio Gambling Compliance database; Viðskiptaráð (2024) (complementary data collection for Iceland).

DNS =  Domain Name System; EGMs = electronic gambling machines.

Tax-related policy changes were similar in the fields of gambling and alcohol. For both commodities, tax rates were in most cases raised. In Denmark, tax rates for online gambling were raised from 20% to 28%/GGR in 2021. In Sweden, the gambling tax rate was increased from 18% to 22%/GGR in 2024 (charity operators are tax exempt). In Finland, the lottery tax was first decreased in 2022 and then increased in 2023. However, this has had little effect on the surplus to state because Finland still operates a monopoly, and all remaining profits are also transferred back to public purposes.

Only the analysis on gambling captured changes in marketing. In all cases, regulations on marketing were tightened or were proposed to be tightened. Most notably in Norway, television advertising of gambling was prohibited in 2022. Other consumer protection measures for gambling were also introduced to further tighten regulations. These included new provisions on limit-setting in Denmark, Finland and Norway, as well as regulations on duty of care practices of operators in Finland and Sweden.

## Discussion

This study has focused on the existence and politics of the so-called Nordic model in the field of control policy for alcohol and gambling. The Nordic model refers to an economic model characterised by state intervention and welfare policy. In previous literature, the Nordic model has been described as an effective way of limiting consumption of harmful commodities, as well as reducing or even preventing harms (Lundberg et al., 2008). Our results have shown many similarities across countries and products, particularly with regard to a strong public health focus and the visibility of the best buys in regulatory approaches. However, we also found differences in terms of policy aims, implementation and outcomes. The main points of divergence related to how countries and policies were placed within a spectrum from more restrictive to more market-oriented. Generally, alcohol policy was more restrictive than gambling policy across the region. This finding was visible in most results of our analysis.

First, our analysis on policy framings showed that, although alcohol policy was governed by the aim of reducing harm by limiting total consumption across all Nordic countries, gambling policy aims were more varied, nuanced and even contradictory. All countries had crime prevention and security-related legislative aims alongside health-related framings for gambling. Iceland, Norway and Sweden also included financial objectives in their legislative aim. Our results align with previous research looking into the legal determinants of health in the gambling field (Wardle et al., 2024; Hörnle et al., 2018). Notably, a recent global analysis of legislative aims (Wardle et al., 2024) found that contradictory aims are common in gambling law, and that this overlap may form an obstacle to effective regulation.

Second, our review of the ministries and authorities responsible for implementing alcohol or gambling policy mostly aligned with the results on policy framings. The health-only framings in alcohol policy across the region were supported by having the locus of responsibility for these policies placed within ministries for health and welfare. The regulatory responsibility shows that, across all Nordic countries, alcohol-related issues appear to be conceptualised primarily as health issues. This unity has only been challenged recently, with Finland investigating whether alcohol should be regulated by the Ministry of Economic Affairs and Employment instead.

Contrary to alcohol, responsibilities for gambling policy reflected the varied legal framings. Responsibility for gambling policy was not placed within ministries of health in any Nordic country. Instead, responsibility was allocated to ministries in charge of finance, taxation, interior, justice or culture. This finding is in line with a prior European review showing that the regulatory responsibility for gambling tends to be allocated to ministries in charge of finance (Marionneau et al., 2018). Research has also shown that the locus of regulatory responsibility tends to depend on the arguments used in the initial legalisation of gambling (Marionneau et al., 2018; Polders, 1997; Sulkunen et al., 2019). For example, if gambling was legalised to prevent criminal involvement, it is natural to place responsibility with a ministry of interior. If gambling was legalised to produce revenue for state, its placement within a ministry in charge of finance becomes logical. However, this initial situating of regulatory responsibility is also likely to have important implications on what kind of policy is prioritised and how the policy area is viewed in public discourse.

Third, our analysis on policy implementation showed that alcohol tends to be more strictly regulated than gambling across the Nordics. The more restrictive implementation of alcohol policy, in comparison to gambling, was particularly visible in a higher prevalence of monopolistic structures, stricter regulations on availability of alcohol products and more encompassing marketing regulations.

Despite experiencing significant turbulence and ongoing political debates across the area (e.g., Iceland, Finland, cf. Lindeman & Karlsson, 2024), alcohol monopolies continue to operate. In contrast, several Nordic gambling monopolies have been dismantled or at least reduced in size in the past 15 years. This was the case for the Danish monopoly Danske Spil in 2012 and the Swedish monopoly Svenska Spel in 2019, and will also be the case for the Finnish monopoly Veikkaus in 2027. In each case, the monopoly has continued existing also in the new license-based system, but the monopoly remit has been reduced to only a small selection of products such as the national lottery or scratch cards (Spångberg & Svensson, 2022; Cisneros Örnberg & Tammi, 2011; Järvinen-Tassopoulos et al., 2024).

A key reason behind the dismantling of monopoly structures has been the competition emanating from cross-border offshore sales of gambling. Although alcohol is also accessible through online sales, this factor alone does not seem to account for a similar pressure to dismantle alcohol monopolies. Rather, domestic policy discourse has intensified, marked by heightened debates and calls for more de-regulation within some of the Nordic countries. As a digital-first commodity (Wardle et al., 2024), cross-border gambling provision is more difficult to regulate, even with existing blocking measures. Dismantling monopolies can therefore also function as a strategy to better control the market. In Sweden and Finland, for example, channelling consumption to the regulated market have been key justifications in the decision to license private overseas providers (Eduskunta, 2021; SOU, 2017).

Fourth, and finally, policy framings, policy responsibilities and policy implementation are visible in policy outcomes. In particular, we found that the more market-oriented policies in Denmark and Finland were also visible as higher prevalence and higher total consumption of alcohol and gambling. Interestingly, despite the stronger public health focus in alcohol policy in comparison to gambling policy, alcohol use was overall more prevalent than gambling across all Nordic countries. This result was visible as higher surplus to state, as well as higher population prevalence. Of course, our results do not allow us to draw causal links between policies and policy outcomes. It is also possible that a baseline high prevalence of alcohol consumption across societies has resulted in strict policy measures and framings for alcohol.

Similarly, some ostensibly more market-oriented policies, such as the introduction of a licensing system or low taxation, may work differently in different contexts. Notably, the effects of taxation on public health are not as clear cut for gambling as for alcohol because higher taxation can also mean higher losses and financial harm to individuals who gamble. Licensing and monopoly systems can in themselves also be inherently different. All monopoly structures are not effective in terms of harm prevention, while some licensing systems may have very high levels of consumer protection (Marionneau et al., 2021).

We also reviewed and compared recent or on-going policy changes in the fields of alcohol and gambling to analyse the directions that policies are taking or are likely to take in the future. This analysis showed that, perhaps surprisingly, the overall direction of alcohol policy appears to be towards gradual step-by-step de-regulation. This is the same direction as in gambling policy some years earlier (Cisneros Örnberg & Tammi, 2011; Aimo et al., 2023). In contrast, in gambling policy, recent and on-going policy changes were towards a more restrictive direction. At face value, this result appears to be somewhat contradictory. However, gambling policy has been, as a rule, more market-oriented in recent years. This may have translated into important and increasingly visible harms (Tran et al., 2024). Increased public health attention to these harmful consequences may therefore have resulted in at least some of the observed restrictions.

In contrast to gambling policy, the Nordic alcohol field has historically been more stable and even static, focusing on set objectives centring around reducing consumption. In recent years, however, the landscape has undergone changes. Denmark has taken a small step towards a more restrictive alcohol policy, focusing on reducing availability for young consumers. A prevention plan, as endorsed by the Danish government and four other political parties, aims to address high alcohol consumption among adolescents and to curb the illegal sale of age-restricted products to underage youth. While these measures have been criticised for being insufficient (Rømer Thomsen et al., 2024), they constitute a step in a new direction.

Current alcohol policy discussions in Iceland and Sweden centre on the politics of external competition to alcohol monopolies. In Iceland, the monopoly is challenged by an unclear legal status of alcohol sales in supermarkets and in online environments. These debates highlight tensions between maintaining public health objectives and addressing emerging market practices that undermine regulatory frameworks (Lindeman & Karlsson, 2024). In Sweden, Systembolaget is similarly pursuing legal action against foreign suppliers targeting Swedish consumers through online sales (Högsta domstolen, 2023). The Swedish alcohol monopoly faces additional competition from farm-based alcohol sales. In February 2024, an official investigation was launched to assess the overall effectiveness of Swedish alcohol policy (Regeringskansliet, 2024). These developments have made alcohol policy a highly political question, with new stakeholders (microbreweries, distilleries, online sales platforms and supermarkets) exerting important influence within the policy landscape.

Significant policy shifts have also recently occurred in Finland where the current right-leaning government has adopted a more market-oriented approach, challenging the public health-oriented foundations of existing policies in favour of economic considerations. These changes are evident in recent alcohol policy reforms, which aim to expand availability and adjust taxation practices. The stated aim of the government programme is to reform Finland's alcohol policy “in a responsible manner to align it more closely with alcohol policies in other European countries” (Valtioneuvosto, 2023). The proposed shift of regulatory responsibility to the Ministry of Economic Affairs and Employment reflects this change in prioritisation and underlying framing of alcohol policy.

This study set out to investigate whether there is a Nordic model for regulating alcohol and gambling. Our results show evidence of a Nordic model in alcohol policy (with the exception of Denmark), but less so for gambling. With respect to alcohol policy, the Nordic model is also showing signs of weakening, particularly in Finland and Sweden. While Nordic countries still share many similarities, they also differ in terms of many policy choices in the fields of both alcohol and gambling. Popular support for monopolies and restrictive policies continues to be strong (Grönroos et al., 2024; WHO Europe, 2025) but on-going changes and global challenges in terms of online provision continue to undermine the effectiveness of national policy. However, as also argued by Andersen et al. (2016), the Nordic model is characterised by constant change in implementation, but stability in overarching principles and aims. This may also apply to control policies in the fields of alcohol and gambling. It is possible that the Nordic model will continue to exist, despite changes in how it is implemented. In our analysis, policy framings suggested a strong commitment to a Nordic model, particularly for alcohol. However, policy implementation varied to a more significant degree. This can result from other, unvoiced, rationales or the politics of competing interests.

Our study was limited by available data sources. Alcohol and gambling are inherently different products, and our focus on best buys was not always applicable to gambling regulation. We were also able to focus only on very top-level outcome indicators because overall monitoring of consumption and harm varies between these fields and even across countries. For example, mortality rates or other harms such as arrests or the range of harm to others could not be included due to the difficulties in obtaining comprehensive statistics on these for gambling. On the other hand, the lack of a gold standard diagnostic measure of alcohol problems, comparable to PGSI in gambling, made it difficult to evaluate effects on public health across countries. We were also not able to provide figures for offshore consumption. Some official figures for cross-border alcohol sales exist but the tracking of offshore sales of gambling is strongly debated and no gold standard measure has been available. Finally, we were not able to retrieve all relevant information, particularly for Iceland. This may be not only a result of linguistic limitations, but also a factor of less comprehensive reporting on alcohol and gambling in Iceland in comparison to other Nordic countries.

Further studies are particularly needed on the kinds of stakeholders that influence policy choices and directions in the Nordic countries. Furthermore, it would be important to extend this type of analysis to other adjoining fields, such as emerging tobacco products (novel nicotine products) or use of nitrous oxide. Similarly, it would be important to compare Nordic control policies to policies within a wider geographical area. It is possible that similarities in the Nordic approach would be more visible compared to other kind of political and policy traditions. Notably, the Baltic countries have recently taken steps in the field of alcohol as well as gambling policy. Estonia and Latvia have intensified their efforts to regulate alcohol consumption through comprehensive strategies and legislative amendments, while Lithuania continues to uphold its earlier reforms (Beekman, 2023). The Baltic countries have also introduced comparatively restrictive gambling advertising policies, compared to the Nordics. Learning from these examples could also be illustrative to the Nordic context.

In conclusion, while we found that the Nordic Model continues to persist, particularly in the field of alcohol control and in terms of policy objectives, this model is challenged by politics and stakeholder pressure. Political will and adaptation are therefore needed if the Nordic model is to endure.

One way forward would be increased inter-sectoral collaboration and learning. Developments in this direction have already occurred in Sweden, with addictive sectors increasingly viewed as a whole under the public health umbrella (Regeringskansliet, 2022). Our results show that many policy questions in alcohol and gambling are similar. For example, current stakeholder pressures and cross-border competition to alcohol monopolies are questions that have, for long, been debated in the gambling field. Similarly, many effective public health policies in the alcohol field are applicable to gambling. To further improve learning and sharing of good practices, inter-sectoral collaboration needs to be global. Many current policy issues are similar across jurisdictions. The Nordic model has the potential to function as an exemplary case to other countries that are debating the need for more public health-oriented regulations on alcohol and gambling. Conversely, the Nordic model may benefit from adaptation to new challenges in a changing global landscape.

Our results also suggest that there is a need to develop gambling-specific recommendations for effective public health regulation. Such “gambling best buys” should particularly focus on effective regulations in the digital environment. As gambling and alcohol markets increasingly expand in the online world, it is crucial that international guidelines adapt to these changes and continue to provide guidelines to other jurisdictions.

## References

[bibr91-14550725251350445] Áfengislög (1989). https://www.althingi.is/lagas/120a/1969082.html

[bibr19-14550725251350445] Áfengislög (2011/86). Act on Trade in Alcohol and Tobacco (English translation). https://www.vinbudin.is/Portaldata/1/Resources/um_atvr/log_og_reglur/Act_on_Trade_in_Alcohol_and_Tobacco__No__86_2011.pdf

[bibr1-14550725251350445] Aimo N. Bassoli M. Marionneau V. (2024). A scoping review of gambling policy research in Europe. International Journal of Social Welfare, 33(3), 659–674.

[bibr2-14550725251350445] Alkoholilaki (2017/1102). https://finlex.fi/fi/laki/ajantasa/2017/20171102

[bibr3-14550725251350445] Alkohollag (2010/1622). https://www.riksdagen.se/sv/dokument-lagar/dokument/svensk-forfattningssamling/alkohollag-20101622_sfs-2010-1622

[bibr4-14550725251350445] Alkoholloven (1989). https://lovdata.no/dokument/NL/lov/1989-06-02-27/KAPITTEL_1#KAPITTEL_1

[bibr5-14550725251350445] Andersen T. M. Holmström B. Honkapohja S. Korkman S. Tson S. H. Vartiainen J. (2007). The Nordic Model. Embracing globalization and sharing risks. Helsinki: Taloustieto.

[bibr6-14550725251350445] Andersen T. M. Schjelderup G. Roine J. Bratsberg B. Roed K. Svarer M. Rosholm M. Takalo T. Toivanen O. Johnsen J. V. Løken K. V. Moene K. (2016). Nordic Economic Policy Review: Whither the Nordic Welfare Model? Nordic Council of Ministers.

[bibr7-14550725251350445] Arpajaislaki. (2001/1047). https://www.finlex.fi/fi/laki/ajantasa/2001/20011047

[bibr8-14550725251350445] Babor T. F. Casswell S. Graham K. Huckle T. Livingston M. Österberg E. Rehm J. Room R. Rossow I. Sornpaisarn B. (2023). Alcohol: No ordinary commodity: Research and public policy (3rd edn). Oxford University Press. 10.1093/oso/9780192844484.001.0001

[bibr9-14550725251350445] Beekman L. (2023) From highs to lows: Alcohol policies in the Baltic Region | NVC. PopNAD https://nordicwelfare.org/popnad/en/artiklar/from-highs-to-lows-alcohol-policies-in-the-baltic-region

[bibr10-14550725251350445] Bloomfield K. Kilian C. Manthey J. Rehm J. Brummer J. Grittner U. (2022). Changes in alcohol use in Denmark during the initial months of the COVID-19 pandemic: Further evidence of polarization of drinking responses. European Addiction Research, 28(4), 297–308. 10.59/000524379 35545059 PMC9254281

[bibr11-14550725251350445] CAN (2023). Alkoholkonsumtionen 2023: Preliminära uppgifter. https://www.can.se/app/uploads/2024/03/alkoholkonsumtionen-2023-preliminara-uppgifter.pdf

[bibr12-14550725251350445] Cisneros Örnberg J. (2009). The Europeanization of Swedish Alcohol Policy. Stockholm University, Faculty of Social Sciences, Centre for Social Research on Alcohol and Drugs (SoRAD).

[bibr13-14550725251350445] Cisneros Örnberg J. Hettne J. (2018) Sweden’s future gambling market: Challenges in law and policies in gambling policies in European welfare states – current challenges and future prospects (pp 197–216). In Egerer M. Marionneau V. Nikkinen J. (Eds). Palgrave/McMillan.

[bibr14-14550725251350445] Cisneros Örnberg J. Tammi T. (2011). Gambling problems as a political framing—safeguarding the monopolies in Finland and Sweden. Journal of Gambling Issues, 26, 110–125. 10.4309/jgi.2011.26.8

[bibr15-14550725251350445] Dahlström S. (2024). Farm sales set the wheels in motion – Could lead to the collapse of Systembolaget. PopNAD. https://nordicwelfare.org/popnad/en/artiklar/farm-sales-set-the-wheels-in-motion-could-lead-to-the-collapse-of-systembolaget/

[bibr16-14550725251350445] Danmarks statistik (2023). Salg af ren alkohol, pr. indb. over 18 år (gns. antal liter). https://www.statistikbanken.dk/statbank5a/SelectVarVal/Define.asp?MainTable=ALKO2&PLanguage=0&PXSId=0&wsid=cftree

[bibr17-14550725251350445] Dómsmálaráðuneytið (2024). Spilahegðun og spilavandi fullorðinna Íslendinga á árinu 2023. https://www.stjornarradid.is/library/02-Rit–skyrslur-og-skrar/Sk%c3%bdrsla%20um%20spilaheg%c3%b0un%20og%20spilavanda.pdf

[bibr18-14550725251350445] Eduskunta (2021). Hallituksen esitys eduskunnalle laiksi arpajaislain muuttamisesta ja siihen liittyviksi laeiksi. https://www.eduskunta.fi/FI/vaski/HallituksenEsitys/Sivut/HE_135+2021.aspx

[bibr20-14550725251350445] Finnish Institute for Health and Welfare (2024a). Alkoholijuomien matkustajatuonti ja verkko-ostaminen 2023. Tilastoraportti 10/2024. https://www.julkari.fi/bitstream/handle/10024/148582/Tr10-2024.pdf?sequence=1&isAllowed=y

[bibr21-14550725251350445] Finnish Institute for Health and Welfare (2024b). Suomalaisten rahapelaaminen 2023. Tilastoraportti 15/2024. https://www.julkari.fi/bitstream/handle/10024/148897/Tilastoraportti%2015%202024.pdf?sequence=5&isAllowed=y

[bibr22-14550725251350445] Finnish Institute for Health and Welfare (2024c). Alkoholijuomien kulutus 2023. Tilastoraportti 39/2024. https://www.julkari.fi/bitstream/handle/10024/149288/Tilastoraportti-39-2024.pdf?sequence=1&isAllowed=y

[bibr23-14550725251350445] Folkehelseinstituttet (2022). Rusmiddellidelser i Norge. https://www.fhi.no/he/folkehelserapporten/psykisk-helse/rusmiddellidelser/?term=#rusmiddellidelser

[bibr24-14550725251350445] Folkehelseinstituttet (2023). Alkohol i Norge. Omsetning og bruk: Alkoholomsetningen i Norge. https://www.fhi.no/le/alkohol/alkoholinorge/omsetning-og-bruk/alkoholomsetningen-i-norge/?term=

[bibr25-14550725251350445] Folkhälsomyndigheten (2023). Tabellsammanställning för Swelogs prevalensundersökning 2021. https://www.folkhalsomyndigheten.se/contentassets/f2e84c88da31465e94b1b94357d5b703/tabellsammanstallning-swelogs-prevalensundersokning-2021.pdf

[bibr26-14550725251350445] Grönroos T. Salonen A. Latvala T. Kontto J. Hagfors H. (2024). Suomalaisten rahapelaaminen 2023: Rahapelaaminen vähentynyt, peliongelma yleistynyt ja suh-tautuminen pelaamiseen muuttunut. Terveyden ja Hyvinvoinnin laitos.

[bibr27-14550725251350445] Högsta domstolen (2023). Mål Nr T 4709-22. https://www.domstol.se/globalassets/filer/domstol/hogstadomstolen/avgoranden/2023/t-4709-22.pdf

[bibr28-14550725251350445] Hörnle J. Littler A Tyson G Padumadasa E Schmidt-Kessen M J Ibosiola D. I. (2018). Evaluation of regulatory tools for enforcing online gambling rules and channelling demand towards controlled offers. European Union Publications Office.

[bibr29-14550725251350445] Jann W. Wegrich K. (2017). Theories of the policy cycle. In Handbook of public policy analysis (pp. 69–88). Routledge.

[bibr30-14550725251350445] Järvinen-Tassopoulos J. Marionneau V. Egerer M. (2024). Strengthening channeling policy: The Finnish approach to protecting domestic online gambling market. Frontiers in Sociology, 8, 1272735. 10.3389/fsoc.2023.1272735 38274838 PMC10809280

[bibr31-14550725251350445] Jónsson R. M. Kristjánsson S. (2013). Alcohol policy and public opinion in Iceland, 1989–2012. Nordic Studies on Alcohol and Drugs, 30(6), 539–549. 10.2478/nsad-2013-0050

[bibr32-14550725251350445] Karlsson T. (2014). Nordic alcohol policy in Europe: The adaptation of Finland’s, Sweden’s, and Norway’s alcohol policies to a new policy framework, 1994–2013. Academic Dissertation.

[bibr33-14550725251350445] Karlsson T. Mäkelä P. Tigerstedt C. Keskimäki I. (2020). The road to the alcohol act 2018 in Finland: A conflict between public health objectives and neoliberal goals. Health Policy, 124(1), 1–6. 10.1016/j.healthpol.2019.10.009 31708165

[bibr34-14550725251350445] Kristjánsson S. Jónsson R. Jensson V. (2023). Alcohol and Tobacco Use in Iceland – where are we heading? PopNAD. https://nordicwelfare.org/popnad/en/artiklar/alcohol-and-tobacco-use-in-iceland-where-are-we-heading/

[bibr35-14550725251350445] Larsen S. Hansen J. L. (2023). Alkohol på Færøerne – forbrug, lovgivning og debat 2023. PopNAD. https://nordicwelfare.org/popnad/artiklar/alkohol-pa-faeroerne-forbrug-lovgivning-og-debat-2023/

[bibr36-14550725251350445] Lasswell H. D. (1956). The decision process: Seven categories of functional analysis. College of Business and Public Administration, University of Maryland.

[bibr37-14550725251350445] Lög um happdrætti (2005 nr. 38 13. Maí) (2005). Retrieved from: https://www.althingi.is/lagas/nuna/2005038.html

[bibr38-14550725251350445] Lindeman M. (2024). Nu skall Finlands alkoholpolitik bli europeisk – till varje pris? PopNAD. https://nordicwelfare.org/popnad/artiklar/nu-skall-finlands-alkoholpolitik-bli-europeisk-till-varje-pris/

[bibr39-14550725251350445] Lindeman M. Karlsson T. (2024) Går Norden mot vin i matbutiker? PopNAD. https://nordicwelfare.org/popnad/artiklar/gar-norden-mot-vin-i-matbutiker/

[bibr40-14550725251350445] Lundberg O. Yngwe MÅ Stjärne M. K. Björk L. Fritzell J. (2008). The Nordic Experience: Welfare States and Public Health (NEWS). Centre for Health Equity Studies (CHESS), Stockholm University & Karolinska Institute.

[bibr41-14550725251350445] Mäkelä P. Warpenius K. (eds.) (2024). Suomalaisten alkoholinkäyttö, juomatavat ja alkoholihaitat. Juomatapatutkimuksen tuloksia. Terveyden ja hyvinvoinnin laitos (THL). Raportti 6/2024.

[bibr42-14550725251350445] Marionneau V. Egerer M. Nikkinen J. (2021). How do state gambling monopolies affect levels of gambling harm? Current Addiction Reports, 8(2), 225–234. 10.1007/s40429-021-00370-y

[bibr43-14550725251350445] Marionneau V. Hellman M. (2020). What is special about gambling? A comparison of public discourse on Finnish state monopolies in rail traffic, gambling, and alcohol. Critical Gambling Studies, 1(1), 40–49. 10.29173/cgs43

[bibr44-14550725251350445] Marionneau V. Nikkinen J. Egerer M. (2018). Conclusion: Contradictions in promoting gambling for good causes. In Gambling policies in European welfare states: Current challenges and future prospects (pp. 297–314). London: Palgrave Mcmillan.

[bibr45-14550725251350445] Matilainen R. (2016). Cultural and social meanings of gambling in Finland and Sweden: A historical perspective. In Random riches (pp. 119–131). Routledge.

[bibr46-14550725251350445] Ministry of Finance (2024). Mail conversation.

[bibr47-14550725251350445] Nationella folkhälsoenkäten (2024). Hälsan på lika villkor? https://www.folkhalsomyndigheten.se/folkhalsorapportering-statistik/om-vara-datainsamlingar/nationella-folkhalsoenkaten/

[bibr48-14550725251350445] Nikkinen J. Marionneau V. (2021). On the efficiency of Nordic state-controlled gambling companies. Nordic Studies on Alcohol and Drugs, 38(3), 212–226. 10.1177/1455072520968024 PMC8899251 35310613

[bibr49-14550725251350445] Nordic Welfare Center (2024). Labour market integration of adults with alcohol and substance use problems in the Nordic countries. Labour market integration of adults with alcohol and substance use problems in the Nordic countries | NVC.

[bibr50-14550725251350445] Norsk Rikstoto (2024). Årsrapport 2023. https://www.rikstoto.no/Nyheter/Aktuelt/2023/Mars/Rapporter

[bibr51-14550725251350445] Norsk Tipping (2024). Års- og bærekraftrapport 2023. https://www.norsk-tipping.no/selskapet/rapporter/nokkeltall

[bibr52-14550725251350445] Örnberg J. C. Ólafsdóttir H. (2008). How to sell alcohol? Nordic alcohol monopolies in a changing epoch. Nordic Studies on Alcohol and Drugs, 25(2), 129–153.

[bibr53-14550725251350445] Pallesen S. Mentzoni R. A. Syvertsen A. Kristensen J. H. Erevik E. K. Morken A. M. (2023). Omfang av penge- og dataspillproblemer i Norge 2022. Universitetet i Bergen.

[bibr54-14550725251350445] Pengespilloven (2022). https://lovdata.no/dokument/NL/lov/2022-03-18-12

[bibr55-14550725251350445] Peters M. D. Marnie C. Colquhoun H. Garritty C. M. Hempel S. Horsley T. Rosholm M. Takalo T. Toivanen O. Johnsen J. V. Løken K. V. Moene K. (2021). Scoping reviews: Reinforcing and advancing the methodology and application. Systematic Reviews, 10(1), 263. 10.1186/s13643-021-01821-3 34625095 PMC8499488

[bibr56-14550725251350445] Polders B. (1997). Gambling in Europe: Unity in diversity. In Eadington W. R. Cornelius J. A. Cornelius J. A. (Eds.), Gambling: Public policies and the social sciences (pp. 65–100). Institute for the Study of Gambling and Commercial Gaming, University of Nevada.

[bibr57-14550725251350445] Regeringskansliet (2022). Regeringens skrivelse 2021/22:213. En samlad strategi för alkohol-, narkotika-, dopnings- och tobakspolitiken samt spel om pengar 2022–2025. https://www.regeringen.se/rattsliga-dokument/skrivelse/2022/03/skr.-202122213/

[bibr58-14550725251350445] Regeringskansliet (2024). Hälsoekonomiska analyser ska ge bättre uppföljning av folkhälsopolitiken. https://www.regeringen.se/pressmeddelanden/2024/02/halsoekonomiska-analyser-ska-ge-battre-uppfoljning-av-folkhalsopolitiken/

[bibr59-14550725251350445] Rømer Thomsen K. Vallentin-Holbech L. Tolstrup J. (2024). A missed opportunity: The recent political agreement on a new tobacco, nicotine and alcohol prevention plan in Denmark. Nordic Studies on Alcohol and Drugs, 41(3), 367–371. 10.1177/14550725231220125 PMC11186446 38903895

[bibr60-14550725251350445] Room R. Cisneros Örnberg J. (2019). Government monopoly as an instrument for public health and welfare: Lessons for cannabis from experience with alcohol monopolies. International Journal of Drug Policy, 74(12), 223–228. 10.1016/j.drugpo.2019.10.008 31698164

[bibr61-14550725251350445] Room R. Tigerstedt C. , et al. (2006). Nordic alcohol policies and the welfare state. In Babor T. F. (Ed.), Alcohol: No ordinary commodity: Research and public policy (pp. 263–279). Oxford University Press.

[bibr62-14550725251350445] Room R. Tigerstedt C. (2007). Nordic alcohol policies and the welfare state. In: Lundborg O. Yngwe M. Å. Stjärne M. K. Björk L. Fritzell J. (Eds.) The Nordic experience: Welfare states and public health (NEWS) (pp. 141–152). Centre for Health Equity Studies (CHESS), Stockholm University & Karolinska Institute.

[bibr63-14550725251350445] Skatteministeriet (2024). Indtaegstliste 2023. https://view.officeapps.live.com/op/view.aspx?src=https%3A%2F%2Fskm.dk%2Fmedia%2Fjtwns13l%2Findtaegtsliste2023.xlsx&wdOrigin=BROWSELINK

[bibr64-14550725251350445] SOU (2017). En omreglerad spelmarknad, p. 30. https://www.regeringen.se/contentassets/29291777554d47e49e717171e4eb5f83/en-omreglerad-spelmarknad-del-1-av-2-kapitel-1-21-sou-201730/

[bibr65-14550725251350445] Spångberg J. Svensson J. (2022). Gambling among 16-year-olds and associated covariates: A Nordic comparison. Scandinavian Journal of Public Health, 50(2), 257–268. 10.1177/1403494820923814 32522086 PMC8873972

[bibr66-14550725251350445] Spelinspektionen (2024). Statistik. https://www.spelinspektionen.se/om-oss/statistik/

[bibr67-14550725251350445] Spellag (2018/1138). https://www.riksdagen.se/sv/dokument-och-lagar/dokument/svensk-forfattningssamling/spellag-20181138_sfs-2018-1138/

[bibr68-14550725251350445] Spilleloven (2020/1303). https://www.retsinformation.dk/eli/lta/2020/1303

[bibr69-14550725251350445] Spillemyndigheden (2022). Survey of the prevalence of gambling and gambling problems in Denmark. https://www.spillemyndigheden.dk/uploads/2022-10/Survey%20of%20the%20prevalence%20of%20gambling%20and%20gambling%20problems%20in%20Denmark%202021.pdf

[bibr70-14550725251350445] Spillemyndigheden (2023). The Gambling Market in Numbers 2023. https://www.spillemyndigheden.dk/uploads/2024-04/The%20Gambling%20Market%20in%20Numbers%202023.pdf

[bibr71-14550725251350445] Statens Institut for Folkesundhed (2024). Alcohol. https://www.sdu.dk/en/sif/forskning/alkohol

[bibr72-14550725251350445] Statistics Iceland (2023). Consumption of alcoholic beverages 1980-2023. https://px.hagstofa.is/pxen/pxweb/en/Samfelag/Samfelag__heilbrigdismal__lifsvenjur_heilsa__1_afengiogreyk/HEI07202.px/table/tableViewLayout2/

[bibr73-14550725251350445] Sulkunen P. Babor T. Cisneros Örnberg J. Egerer M. Hellman M. Livingstone C. Marionneau V. Nikkinen J. Orford J. Room R. Rossow I. (2019). Setting Limits: Gambling, Science, and Public Policy. University Press. 10.1111/add.15241 33084199

[bibr74-14550725251350445] Sulkunen P. Babor T. F. Cisneros Örnberg J. Egerer M. Hellman M. Livingstone C. Marionneau V. Nikkinen J. Orford J. Room R. Rossow I. (2021). Setting limits: Gambling, science and public policy—summary of results. Addiction, 116(1), 32–40. 10.1111/add.15241 33084199

[bibr75-14550725251350445] Thompson C. F. (2025). Comparing gambling policy evolution in Denmark, Finland, Norway, and Sweden. International Journal of Public Administration, 48(5-6), 395–406. 10.1080/01900692.2025.2470760

[bibr76-14550725251350445] Tigerstedt C. (2001). The dissolution of the alcohol policy field: Studies on the Nordic countries. University of Helsinki.

[bibr77-14550725251350445] Tran L. T. Wardle H. Colledge-Frisby S. Taylor S. Lynch M. Rehm J. Volberg R. Marionneau V. Saxena S. Bunn C. Farrell M. Degenhardt L. (2024). The prevalence of gambling and problematic gambling: a systematic review and meta-analysis. The Lancet Public Health, 9(8), E594–E613. 10.1016/S2468-2667(24)00126-9 39025095

[bibr78-14550725251350445] Trolldal B. (2023). Alkoholkonsumtionen i Sverige 2001–2022. Centralförbundet för alkohol- och narkotikaupplysning (CAN), Rapport 221.

[bibr79-14550725251350445] Ugland T. (2002). Policy re-categorization and integration. Europeanization of Nordic alcohol control policies. Department of Political Science, University of Oslo.

[bibr80-14550725251350445] Valtioneuvosto (2023). Vahva ja välittävä Suomi: Pääministeri Petteri Orpon hallituksen ohjelma. https://valtioneuvosto.fi/hallitukset/hallitusohjelma#/

[bibr81-14550725251350445] Veikkaus (2024). Vuosi ja vastuullisuusraportti. https://cms.veikkaus.fi/sites/default/files/content/assets/vuosikertomus/2023/Veikkaus_Vuosi_ja_vastuullisuusraportti_2023.pdf

[bibr82-14550725251350445] Viðskiptaráð (2024). Veðjað á rangan hest: Umgjörð veðmálastarfsemi á Íslandi. https://vi.is/skodanir/vedjad-a-rangan-hest

[bibr83-14550725251350445] Vixio Gambling Compliance (2021) (database). https://www.vixio.com/gambling-compliance (under license)

[bibr84-14550725251350445] Wardle H. Degenhardt L. Marionneau V. Reith G. Livingstone C. Sparrow M. Tran L. T. Biggar B. Bunn C. Farrell M. Kesaite V. Poznyak V. Quan J. Rehm J. Rintoul A. Sharma M. Shiffman J. Siste K. Ukhova D. ,K Saxena S. (2024). The lancet public health commission on gambling. The Lancet Public Health, 9(11), e950–e994. 10.1016/S2468-2667(24)00167-1 39491880

[bibr85-14550725251350445] World Health Organization (2011). Global status report on noncommunicable diseases 2010. https://www.who.int/nmh/publications/ncd_report_full_en.pdf

[bibr86-14550725251350445] World Health Organization (n.d.). Global information system on alcohol and health (GISAH). https://www.who.int/data/gho/data/themes/global-information-system-on-alcohol-and-health

[bibr87-14550725251350445] World Health Organization. Regional Office for Europe (2025). Nordic alcohol monopolies: understanding their role in a comprehensive alcohol policy structure and public health significance. World Health Organization. Regional Office for Europe. https://iris.who.int/handle/10665/380344

